# Influence of Human Papillomavirus on Alveolar Bone and Orthodontic Treatment: Systematic Review and Case Report

**DOI:** 10.3390/healthcare10040624

**Published:** 2022-03-26

**Authors:** Oana Almășan, Ioana Duncea, Andreea Kui, Smaranda Buduru

**Affiliations:** Prosthetic Dentistry and Dental Materials Department, Iuliu Hatieganu University of Medicine and Pharmacy, 32 Clinicilor Street, 400006 Cluj-Napoca, Romania; ocrisst@gmail.com (O.A.); andreeakui@gmail.com (A.K.); smarandabudurudana@gmail.com (S.B.)

**Keywords:** papillomavirus infection, orthodontics, biomechanics, tooth mobility

## Abstract

Background: As the human papillomavirus (HPV) infections are detected in healthy oral mucosa as well as in oral lesions, dental practitioners have an important role in detecting any possible lesions that might be caused by this virus. Therefore, the aim of this study was to investigate the outcomes of orthodontic treatments and HPV infections and to report a rare case of ongoing orthodontic treatment superposed on an HPV infection. Methods: An electronic English literature research of the articles published between the years 2011–2021 was conducted between December 2021–February 2022, accessing PubMed, Web of Science, Embase, Scopus, and Google Scholar. The terms “HPV”, “orthodontics”, “orthodontic treatment”, “tooth movement”, “tooth mobility”, and “malocclusion” were searched. The following inclusion criteria were pursued: articles published in English language; studies reporting HPV infection in subjects with past or ongoing orthodontic treatment; and case reports of subjects with HPV and orthodontic treatment. Exclusion criteria were: articles in languages other than English, studies related to malignancies other than HPV and orthodontic treatment; and studies reporting patients with HPV and no orthodontic treatment. Results: Following the systematic review, which includes six papers, a case of orthodontic treatment superposed on a HPV infection is presented. Conclusion: Incumbent, postponed HPV infection on an ongoing orthodontic treatment might affect treatment outcome and patient compliance.

## 1. Introduction

Human papillomavirus (HPV) infections have become more remarkable during the last years, with oropharyngeal manifestations that have to be considered when planning a complex dental treatment plan, especially when the infection occurs during the treatment period. There are more than 200 different HPV genotypes with high or low risk of malignancy [1,2]. Oral squamous papilloma is one of the oral cavity lesions that manifests as a verrucous or papillary exophytic mass [3]. HPV types 6 and 11 are responsible for benign lesions, and type 16 and 18 are responsible for dysplasia [4].

However, new perspectives have been developed for the prevention of these infections by the application of HPV testing technologies and vaccines [5]. There are many different sub-types of HPVs, the majority being asymptomatic and resolving spontaneously within two years [6]. The World Health Organization’s recommends the use of human papillomavirus vaccines as a national immunization program [7]. HPV infection with high-risk types 16 and 18 has been widely reported as a prominent mechanism behind the development of squamous cell carcinoma (SCC) of the oropharynx [8]. HPV is responsible for more than 5% of cancers worldwide, oropharyngeal squamous cell carcinomas and cervical cancers being reported [9], with leading HPV genotypes being HPV 16, 52, 58, 53, 56, and 81 [10]. A subset of oropharyngeal squamous cell carcinoma is associated with human papillomavirus infection, particularly with high-risk type 16 (HPV-16) [11]. Treatment options vary from application of ointments to cryotherapy and surgical removal using lasers, electro surgery, and curettage [12]. 

HPV infections are detected in healthy oral mucosa as well as in oral lesions [13], and therefore, the dentist has an important role in the inspection and palpation of the oral tissues. Oral healthcare should be thorough supervised during dental appointments. Contrary to other viral infections, no treatment is provided for HPV oral lesions, with the management of these including the patient’s follow-up and the periodic probation of the immune system [14]. Surgical treatment of some lesions might be accompanied by the application of low-level laser therapy (LLLT) protocols [15]. However, the surgical removal of the lesion does not guarantee the eradication of the infection since the DNA of the virus could persist in the healthy mucosa [16]. Therefore, the HPV vaccine should be considered, as it is more reliable in preventing the disease than curing it. It has been shown that there is no positive correlation between HPV and the severity of periodontal lesions [17]. When considering dental follow up of patients with complete dentures, it is stated that it may help in monitoring the appearance of possible malignant oral lesions [18]. HPV may be found in the oral cavity of patients with dentures; therefore, HPV-associated diseases, such as oral cancer and other oral lesions, may develop [19]. It has been shown that in subjects with HPV-positive tumors, there has been higher mean alveolar bone loss [20]. Tooth mobility, as a result of alveolar bone and periodontal ligament loss, was associated with an increased risk of HPV-negative oral SCC [21].

Human papillomavirus is rare in children and patients with orthodontic treatment need. Usually, orthodontic treatment is not initiated in cases with positive HPV infection, but when the infection is discovered, by the presence of oral condyloma, warts, or papilloma, orthodontic treatment has already been initiated. There are a few publications worldwide focused on the of HPV infection in subjects with ongoing orthodontic treatment since orthodontics is contraindicated in subjects with malignancies. 

To the best of our knowledge, there has not yet been published a paper related to the possible impact of the genital HPV-58 infection on the outcomes of orthodontic treatments. Therefore, the aim of this study was to review the English literature related to human papillomavirus infections in subjects with ongoing orthodontic treatment and to report a rare case of HPV infection. 

## 2. Materials and Methods

This systematic review was performed in accordance with the recommendations of the “Preferred Reporting Items for Systematic Reviews and Meta-Analyses Protocols (PRISMA) statement” [22].

### 2.1. Information Sources

A structured search was conducted (between December 2021–February 2022) on articles published between the years 2011–2021, accessing PubMed, Web of Science, Embase, Scopus, and Google Scholar databases. In addition, a handsearching of the reference lists of included studies or relevant reviews was performed.

### 2.2. Search Strategy

The terms “HPV”, “orthodontics”, “orthodontic treatment”, “tooth movement”, “tooth mobility”, and “malocclusion” were searched in combination with the Boolean operators “AND” and “OR” All references were imported and organized in the bibliographic software Mendeley^®^ (Mendeley Software, London, UK).

### 2.3. Selection of Articles

The following inclusion criteria were pursued: (1) articles published in English language; (2) studies reporting HPV infection in subjects with past or ongoing orthodontic treatment; and (3) case reports of subjects with HPV and orthodontic treatment. Exclusion criteria were: (1) articles in languages other than English; (2) studies related to malignancies other than HPV and orthodontic treatment; and (3) studies reporting patients with HPV and no orthodontic treatment (Table 1). 

### 2.4. Data Collection

All the eligible citations imported into the bibliography were checked, and all the duplicates were removed. Two reviewers carried out the evaluations independently. For the assessment of each publication, Excel (Microsoft Office 2019^®^, MS, Redmond, WA, USA) spreadsheets were compiled. This way, data were extracted using a standardized form, which included the following information: (1) authors’ names and publication year; (2) study design; (3) aim of the study; (4) methodology; (5) key findings; and (6) conclusions. Afterwards, both authors compared their assessments and confirmed the data on the basis of the compiled spreadsheets. Both researchers compared their assessments and confirmed the data. When in doubt regarding the study data, the two researchers resolved disagreements by discussion, or a third researcher solved discrepancies.

## 3. Results

From the articles published between the years 2011–2021, the terms “HPV and orthodontics” comprised 799 articles in Google Scholar, 192 articles in Scopus, 12 articles in Embase, 10 articles in PubMed, and 1 article Web of Science; “HPV and orthodontic treatment” comprised 235 articles in Google Scholar, 121 articles in Scopus, 2 articles in Embase, 1 article Web of Science, and 1 article in PubMed; “HPV and tooth movement” comprised 563 articles in Google Scholar, 80 articles in Scopus, 1 article in Embase, and 1 article Web of Science; “HPV and tooth mobility” comprised 309 articles in Google Scholar, 29 articles in Scopus, 3 articles in Embase, 3 articles in PubMed, and 3 articles in Web of Science; and “HPV and malocclusion” comprised 97 articles in Google Scholar, 25 articles in Scopus, and 3 articles in Embase (Table 2). 

**Figure 1:**
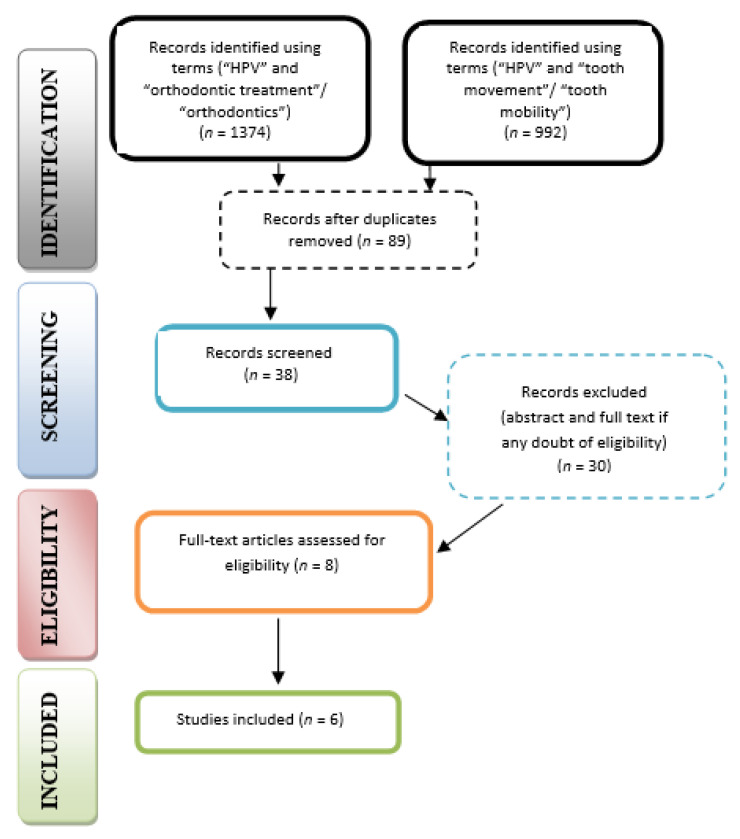
PRISMA flow diagram for research stages; literature search showed the following articles that reported a relationship between HPV and orthodontic treatment (Table 3).

After excluding the duplicates, 89 records were included for screening. From the initial literature review, 11 articles were identified, which met the inclusion criteria. The remaining eight articles were checked for eligibility by the full-text review, and six full-text articles were selected (Figure 1).

### 3.1. Case Series and Case Report Studies

Out of the six references included in this systematic review, five were case presentations. Santos-Silva et al. (2011) [24] published a paper that aimed to describe a series of cases of nonsmoking and nondrinking young patients diagnosed with tongue squamous cell carcinoma and who also recently received orthodontic treatment or evaluation. While the HPV could not be excluded in the history of these patients, the authors emphasized the importance of malignant lesions screening, as the incidence of such lesions in this segment of population seems to be increasing.

Noonan et al. (2017) presented a series of seven cases of gingival papillary keratosis with unknown etiology. The lesions were bilateral and symmetric, characterized by yellow-white plaques. While the authors did not exclude a HPV infection, the authors suggested that identification of additional patients diagnosed with such lesions may help in the understanding of their etiology [25]. 

Henn et al. (2014) presented a case of condyloma acuminatum lesion on a HIV-positive patient (undergoing an orthodontic treatment) and HPV induced. For this case, the treatment plan included surgical removal and chemical cauterization using trichloroacetic acid (TA). The authors emphasized the importance of correct diagnosis and planning for HPV-induced lesions, as there is a high risk for recurrences [26]. The use of trichloroacetic acid (TA) was investigated also by Moine and Gilligan. (2018) in a case report of a 13-year-old patient suffering from localized juvenile spongiotic hyperplasia (LJSGH) [27]. The authors concluded that TA could be a safe, non-invasive alternative for the treatment of lesions, such as LJSGH.

Magalhaes et al. (2016) presented a case of oral squamous cell carcinoma on an 8-year-old patient undergoing orthodontic treatment. Histopathological exam was p16-positive in a patchy pattern, which is suggestive of HPV. The lesions were located in the gingiva and alveolar ridge, a common location for this demographic group; the post-operative evolution was without events, and the patient was considered disease free at 16 months after surgical resection [28].

### 3.2. Case-Control Study

Schott et al. (2019) [23] conducted a research based on a questionnaire aiming to investigate women’s personal history of orthodontic care, long-term satisfaction, as well as adherence to dental and gynecological screening. The data gathered from 233 participants suggested that women with orthodontic treatment in childhood were more concerned regarding prevention strategies in adulthood, which meant that compliant behavior in this context might be established in childhood.

Further, we will present a case-report of an HPV infection interposed with an orthodontic treatment.

### 3.3. Case Report

**Figure 2:**
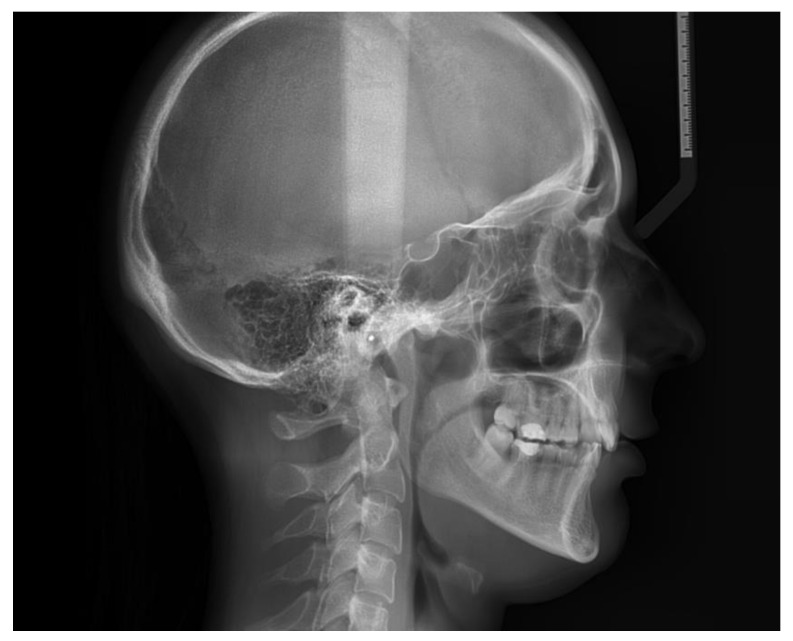
Lateral cephalogram.

**Figure 3:**
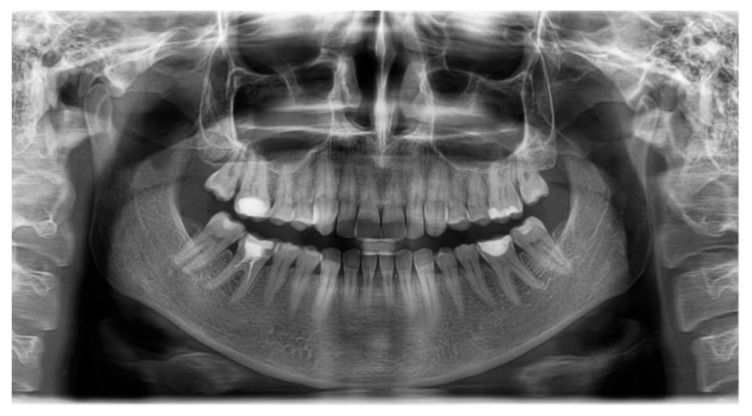
Initial panoramic radiograph.

A 25-year-old female, with a skeletal class II relationship and who had crowding on both arches, seeking orthodontic treatment, presented to our clinic with the main complaint of crowding in both arches (Figure 2, Table 4). The patient showed no signs or symptoms of temporomandibular disorders, no periodontal disease, and no history of medical problems. Periodontal examination showed a pink-colored gingiva with no signs of swelling, bleeding, or tenderness. Clinical assessment of tooth mobility and instrumental mobilometry revealed grade 0 physiological mobility with no signs of ankylosis, gingivitis, or periodontal disease. Radiologic examination showed normal bone height and a thin periodontal biotype (Figure 3). The treatment objective was to address the malocclusion, to improve the crowding, and to level the occlusal plane. Treatment progress: no tooth extractions for space gaining were performed. Occlusal plane was corrected by intrusion of upper molars using skeletal anchorage. Arches were aligned using 0.22 slot metallic brackets. Because no surgical aiding procedures for shortening treatment time were practiced, low forces were applied using light orthodontic wires. 

Orthodontic treatment was initiated before the onset of the SARS-CoV-2 pandemic. At that time, the patient did not have the HPV or SARS-CoV-2 infection and followed her regular orthodontic appointments at about 4–6 weeks. After the pandemic onset, which comprised also a period of two months of closed dental offices in our country (mid-March to mid-May 2020), treatment visits became rare, about 8–10 weeks, and the patient missed a few appointments. Protocols for treating patients during the pandemic have been continuously updated. Risk assessment of the pandemic situation has to be adhered [29], and the new dental guidelines related to treating patients should be honored according to the office‘s location [30], emergencies being admissive and other dental procedures being postponed. 

During the above mentioned period, the patient failed to follow a few appointments, which usually were scheduled monthly, due to the overlap of a genital HPV infection (May 2020). The patient achieved a genital HPV 58 infection, which was diagnosed by real-time PCR multiplex technology, a test that allows simultaneous detection of 19 HPV high-risk types (16, 18, 26, 31, 33, 35, 39, 45, 51, 52, 53, 56, 58, 59, 66, 68, 69, 73, 82) and 9 low-risk HPV types (6, 11, 40, 42, 43, 44, 54, 61, 70) as well as intern control. Intern control inspected PCR reaction to each sample. Every reaction was monitored by using six positive intern controls and two negative intern controls. Biopsy of the cervix revealed cervical intraepithelial neoplasia grade 1. Immunohistochemistry using Benchmark Gx Ventana-Roche platform showed intense inflammatory cervicitis and pavement epithelium with nuclear atypia, suggestive for a low-grade squamous intraepithelial lesion (LSIL). 

**Figure 4:**
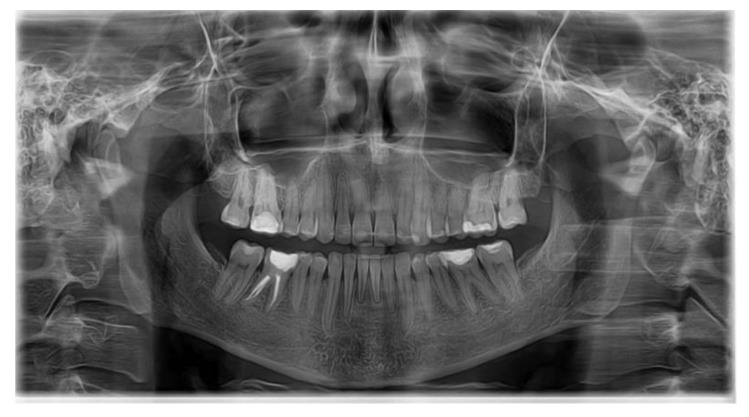
Final panoramic radiograph.

No signs or symptoms of oral HPV were noted. During the infection and treatment for HPV, the patient missed three appointments due to her medical condition; whilst she had her surgery, the orthodontic treatment time was prolonged and tooth movement difficult; in addition, the response of the periodontium to the orthodontic forces was impracticable and incompliant. An increase of tooth mobility was observed clinically. Unanticipated difficulties, such as debonded brackets, space appearance, broken wires, lower incisor proclination, lost springs, and exposed end of wire, occurred. In spite of the HPV onset, leveling and alignment of the arches were acceptable, and the occlusal plane was successfully corrected. However, no significant periodontal pathology (severe alveolar bone loss, gingival recession, loss of tooth vitality) occurred at the end of the treatment (Figure 4).

## 4. Discussion

As previously stated, HPV infections became more frequent among young patients in an oropharyngeal manifestation, which have to be considered when planning a complex treatment plan, especially an orthodontic therapy. While this systematic review aimed to identify any correlations between ongoing orthodontic treatments along with their outcomes and HPV infection, the literature suggests a lack of such data so far. Out of the six articles included, five were case reports or case series of different oral lesions superposed with an HPV infection (confirmed or not). These studies do not concentrate whatsoever on the effects of the orthodontic therapy but rather on the diagnostic, treatment, and prognostic of the oral manifestations presented. In addition, an interesting approach was identified in the paper published by Schott et al., which investigated, through a questionnaire form, 233 women’s personal history of orthodontic care, long-term satisfaction, as well as adherence to dental and gynecological screening [23]. Based on the findings, the authors suggested that it might be strong association between the level of interest towards orthodontic treatments in childhood and the level of prevention in adulthood along with the level of education, making referrals to HPV infection as well.

Orthodontic tooth movement is consequent of an alternation of bone resorption and bone formation, which takes place yearlong [31], and could reach a period of two or more years [32,33], being stimulated by remodeling of the periodontal ligament and the alveolar bone and these remodeling processes of the ligaments and alveolar bone being accompanied by an inflammatory process [34]. 

Treating class 2 malocclusion is challenging relating to the dental and skeletal problems as regards achieving stable results and sparing the periodontal tissues. This prolonged time interval could influence the teeth mobility as well as the support tissues: the periodontal ligament and alveolar bone. Associated pathology or general disease may also have a major role on tooth movement and the response to orthodontic forces. Prolonging the appliance activation period for better healing and optimal stability could improve prevention of alveolar bone loss or tooth ankylosis. Another aid in delivering a safe treatment strategy is to shorten the treatment time. Procedures that could shorten the treatment time have been described, such as distraction osteogenesis [35], corticotomies of the alveolar process [36], osteoperforation [37,38,39], corticision [40], piezocision [41], vibrational forces [42], and low-dose laser application [43]. The amount and rate of tooth movement are hinged to the biological response of the applied forces and the amount of bone turnover [38]. 

The HPV infection of the patient was discovered due to the appearance of a characteristic macroscopic wart in the genital area. Cervical cytology by Pap test revealed atypical squamous cells of undetermined significance (ASC-US). After that, orthodontic treatment became subsidiary. 

Deferring orthodontic treatment until the cure of the HPV infection will prolong the treatment period, with possible repercussions. Ankylosis, root resorption, and marginal bone loss can be associated with prolonged orthodontic treatment time due to the superposition of the HPV infection. The increased treatment time may also lead to unsatisfactory treatment outcome, as also reported by Umeh et al. [44]. Shortening treatment time would aid in avoiding problems that might occur during orthodontic treatment, especially in case of a general disease superposition, which might affect final outcome. Likewise, iatrogenic disorders might occur during orthodontic treatment if forces are heavy or treatment time prolonged: root resorption (inflammatory or of unknown etiology), alveolar bone loss, increased tooth mobility, and periodontal pockets. Shortening treatment time would aid in avoiding problems that might occur during orthodontic treatment, achieving the treatment goal without affecting the treatment outcome. 

Human papillomavirus was first described as “human warts virus” implicated in the pathogenesis of laryngeal papillomatosis and genital warts, also being described as having a potential carcinogenetic role [45]. In as far as having such an important role in developing malignancies of the upper digestive tract, it is interesting to note a possible influence also on ongoing treatments, especially those who induce a modification of the cellular response to bone resorption and bone remodeling, as an orthodontic treatment does. It may play a role in the pathogenesis of cell turnover. However, in the literature are a few reports linked to orthodontics and HPV infection. A higher prevalence of HPV infection is reported in the anogenital region compared to the oral cavity [46], with women presenting the highest disease burden [47]. The management of the disease depends upon its evolution, which can be spontaneous regression or high-grade dysplasia or invasive carcinoma [48]. 

Routine screening of patients needing orthodontic treatment is not performed although screening for HPV infection is extremely important. We want to emphasize this aspect and draw attention on this subject, as such rare cases can occur by chance. It is essential to know about the etiology, prevention, and treatment of HPV related tooth mobility in as much as HPV is a risk factor for increasing tooth mobility in patients with ongoing orthodontic treatment. Screening for HPV is extremely important, as HPV genotyping significantly improves detection rate of high-grade cervical intraepithelial lesions [49]. It is shown that HPV status of tumors has a relationship with response to treatment and survival rates [50]; therefore, the major role of the dentist in prevention and patient education must be emphasized. 

Unfortunately, there are no available guidelines regarding screening for HPV-related malignancies in other anatomic sites [51]. In light of these findings, knowing the effects of HPV infection on oral cavity, vaccines should be considered and their preventive role discussed with the patient. A good alternative would be the administration of the vaccine by dentists or orthodontists. Therefore, discussions about HPV vaccinations in the dental practice are welcome [52]. 

Future recommendations: screening children and young adolescences for HPV is an area of great interest, which necessitates future research since HPV may relate to the treatment encroachment. Complete oral examinations as a routine in orthodontic treatment may contribute to the early diagnosis of HPV-related symptoms. 

A limitation of this study may be the absence of any oral manifestations of the HPV infection and the lack of communication between patient and practitioner due to the embarrassment associated with HPV infection. 

## 5. Conclusions

Based on the findings obtained through the systemic review, literature suggests that HPV infections are increasing in young patients, and the oral manifestations might be identified via intraoral examinations by dental practitioners of any specialization, including orthodontics. 

Incumbent, postponed ongoing orthodontic treatment because of HPV infection might affect treatment outcome and patient compliance. Treatment time lengthens, and unwanted impediments can come into existence. Orthodontists should treat patients while taking into account superposed disease that might influence treatment outcome by prolonging treatment time and influencing the responding support tissues. 

## Figures and Tables

**Table 1:** Inclusion and exclusion criteria.

Criterion	Inclusion	Exclusion
Time period	Publications available between January 2011 and December 2021	All publications published before January 2011
Language	English	Non-English
Type of articles	Publications reporting HPV infection with past or ongoing orthodontic treatment; case reports of subjects with HPV and orthodontic treatment. Publications for which full text is available	Studies related to malignant lesions other than HPV and orthodontic treatments; Research only focusing on HVP oral lesions without orthodontic treatments

**Table 2:** English literature research of articles.

English Literature Research of Articles Published between 2011–2021	HPV and Orthodontics	HPV and Orthodontic Treatment	HPV and Tooth Movement	HPV and Tooth Mobility	HPV and Malocclusion
PubMed	10	1	0	3	0
Web of Science	1	1	1	3	0
Embase	12	2	1	3	3
Scopus	192	121	80	29	25
Google Scholar	799	235	563	309	97

**Table 3:** Selected articles who met the inclusion criteria.

Authors	Methods	Orthodontic Treatment	HPV Relationship	Results
Schott S. et al., 2019 [23]	Case-control	In the past (childhood)	omen with orthodontic treatment in the past were more prone to prevention strategies for HPV in adulthood	“…concordance with the argumentation that cervical dysplasia occurs more frequently among lower income and education levels; women without orthodontic treatment was significant less aware of prevention strategies such as the HPV vaccination”.
Santos-Silva A.R. et al., 2014 [24]	Case reports (3 cases)	In the past (recently)	Tongue squamous cell carcinoma, HPV uncertain	“…full oral examinations, including the entire oral mucosa, as routine in orthodontia could significantly contribute to the early diagnosis of oral cancer”.
	Case 1: 21-year-old woman	Final stage of orthodontic treatment	SCC (squamous cell carcinoma)–biopsy	
	Case 2: 34-year-old man	Completed orthodontic treatment 4 years earlier	SCC–biopsy	
	Case 3: 29-year-old woman	After an initial orthodontic evaluation, approximately 40 days before	SCC–biopsy	
Noonan V.L. et al., 2017 [25]	Case report, 17 year-old male, Caucasian	Orthodontic retainer nightly/	Possible HPV etiology, although uncertain	“…the lesions presented exclusively in patients in the second decade localized to the anterior maxillary attached gingiva sparing the marginal gingiva and stopping abruptly at the mucogingival junction”.
Henn IW et al., 2014 [26]	Case report, 37 year-old male	Yes, ongoing	HPV infection	“Oral condyloma acuminatum was noted in the patient in the form of multiple lesions verrucous, and staining with variable sizes”.
Moine L., Gilligan G., 2018 [27]	Case report, 13 year-old male	Yes, ongoing	Possible HPV etiology, although uncertain	Localized juvenile spongiotic gingival hyperplasia (LJSGH) was treated with trichloroacetic acid (TA) after a conventional surgical treatment. TA could be a safe alternative and a non-invasive technique to treat lesions associated to LJSGH.
Magalhaes M.A. et al., 2016 [28]	Case report, 8-year-old male	Yes, ongoing	Squamous cell carcinoma, with positive staining for p16 in a patchy pattern suggestive of HPV	This rare case of squamous cell carcinoma was located in the gingiva and alveolar ridge, a common location for this demographic group; the post-operative evolution was without events, and the patient was considered disease free at 16 months after surgical resection.

**Table 4:** Cephalometric tracing.

Measurements	Result	Mean	S.D.	Meaning
SNA	80.03	81.08	3.7	Normal A-P position of the maxilla
SNB	75.40	79.17	3.8	Normal A-P position of the mandible
ANB	2.46	4.63	1.8	skeletal class II
FMA	26.32	29.63	3.0	Hypodivergent facial pattern
Gonial angle	123.44	124.31	5.4	Normal gonial angle
APDI	74.22	85.74	4.0	Skeletal class II
A to N-Perp (FH)	−2.58	0.4	2.3	Retruded maxilla
B to N-Perp (FH)	−12.06	−3.5	2.0	Retruded mandible
Pog to N-Perp (FH)	−9.14	−1.8	2.5	Retruded chin point
FH to AB	76.26	81	3.0	Skeletal class II
A-B to mandibular plane	77.41	69.3	2.5	Large angle
Wits appraisal	5.61	−2.74	0.3	Skeletal class II
Overjet	4.79	2	2.0	Large overjet
Overbite	2.46	2	2.0	Normal overbite
U1 to FH	100.88	113.8	6.4	Retroclined upper incisor
U1 to SN	93.59	105.28	6.6	Retroclined upper incisor
U1 to UOP	70.28	55	4.0	Retroclined upper incisor
IMPA	80.50	91.62	3.2	Retroclined lower incisor
L1 to LOP	74.77	66	5.0	Retroclined lower incisor
Interincisal angle	152.29	128	5.3	Uprighted interincisal angle
Cant of occlusal plane	5.66	9.3	3.8	Normal occlusal plane angle
U1 to NA(mm)	0.10	4	3.0	Retruded upper incisor
U1 to NA(deg)	13.55	22	5.0	Retroclined upper incisor
L1 to NB(mm)	1.5	4	2.0	Retruded lower incisor
L1 to NB(deg)	9.52	25	5.0	Retroclined lower incisor
Upper incisal display	3.17	2.5	1.5	Normal incisal display
Upper lip to E-plane	−3.42	0	2.0	Retruded upper lip
Lower lip to E-plane	−2.22	0	2.0	Retruded lower lip
Nasolabial angle	114.84	95	5.0	Retruded lip
Extraction Index	159.69	153.8	7.8	Normal
